# Exploring Patient Journey Mapping and the Learning Health System: Scoping Review

**DOI:** 10.2196/43966

**Published:** 2023-02-27

**Authors:** Amanda L Joseph, Helen Monkman, Andre Kushniruk, Yuri Quintana

**Affiliations:** 1 School of Health Information Science University of Victoria Victoria, BC Canada; 2 Homewood Research Institute Guelph, ON Canada; 3 Division of Clinical Informatics Beth Israel Deaconess Medical Center Boston, MA United States; 4 Harvard Medical School Boston, MA United States

**Keywords:** patient journey map, journey map, patient health information, learning health system, learning health care system, delivery of health care, service delivery, scoping review, health informatics, user experience, data integration

## Abstract

**Background:**

Journey maps are visualization tools that can facilitate the diagrammatical representation of stakeholder groups by interest or function for comparative visual analysis. Therefore, journey maps can illustrate intersections and relationships between organizations and consumers using products or services. We propose that some synergies may exist between journey maps and the concept of a learning health system (LHS). The overarching goal of an LHS is to use health care data to inform clinical practice and improve service delivery processes and patient outcomes.

**Objective:**

The purpose of this review was to assess the literature and establish a relationship between journey mapping techniques and LHSs. Specifically, in this study, we explored the current state of the literature to answer the following research questions: (1) Is there a relationship between journey mapping techniques and an LHS in the literature? (2) Is there a way to integrate the data from journey mapping activities into an LHS? (3) How can the data gleaned from journey map activities be used to inform an LHS?

**Methods:**

A scoping review was conducted by querying the following electronic databases: Cochrane Database of Systematic Reviews (Ovid), IEEE Xplore, PubMed, Web of Science, Academic Search Complete (EBSCOhost), APA PsycInfo (EBSCOhost), CINAHL (EBSCOhost), and MEDLINE (EBSCOhost). Two researchers applied the inclusion criteria and assessed all articles by title and abstract in the first screen, using Covidence. Following this, a full-text review of included articles was done, with relevant data extracted, tabulated, and assessed thematically.

**Results:**

The initial search yielded 694 studies. Of those, 179 duplicates were removed. Following this, 515 articles were assessed during the first screening phase, and 412 were excluded, as they did not meet the inclusion criteria. Next, 103 articles were read in full, and 95 were excluded, resulting in a final sample of 8 articles that satisfied the inclusion criteria. The article sample can be subsumed into 2 overarching themes: (1) the need to evolve service delivery models in health care, and (2) the potential value of using patient journey data in an LHS.

**Conclusions:**

This scoping review demonstrated the gap in knowledge regarding integrating the data from journey mapping activities into an LHS. Our findings highlighted the importance of using the data from patient experiences to enrich an LHS and provide holistic care. To satisfy this gap, the authors intend to continue this investigation to establish the relationship between journey mapping and the concept of LHSs. This scoping review will serve as phase 1 of an investigative series. Phase 2 will entail the creation of a holistic framework to guide and streamline data integration from journey mapping activities into an LHS. Lastly, phase 3 will provide a proof of concept to demonstrate how patient journey mapping activities could be integrated into an LHS.

## Introduction

### What Is a Journey Map?

**Figure 1:**
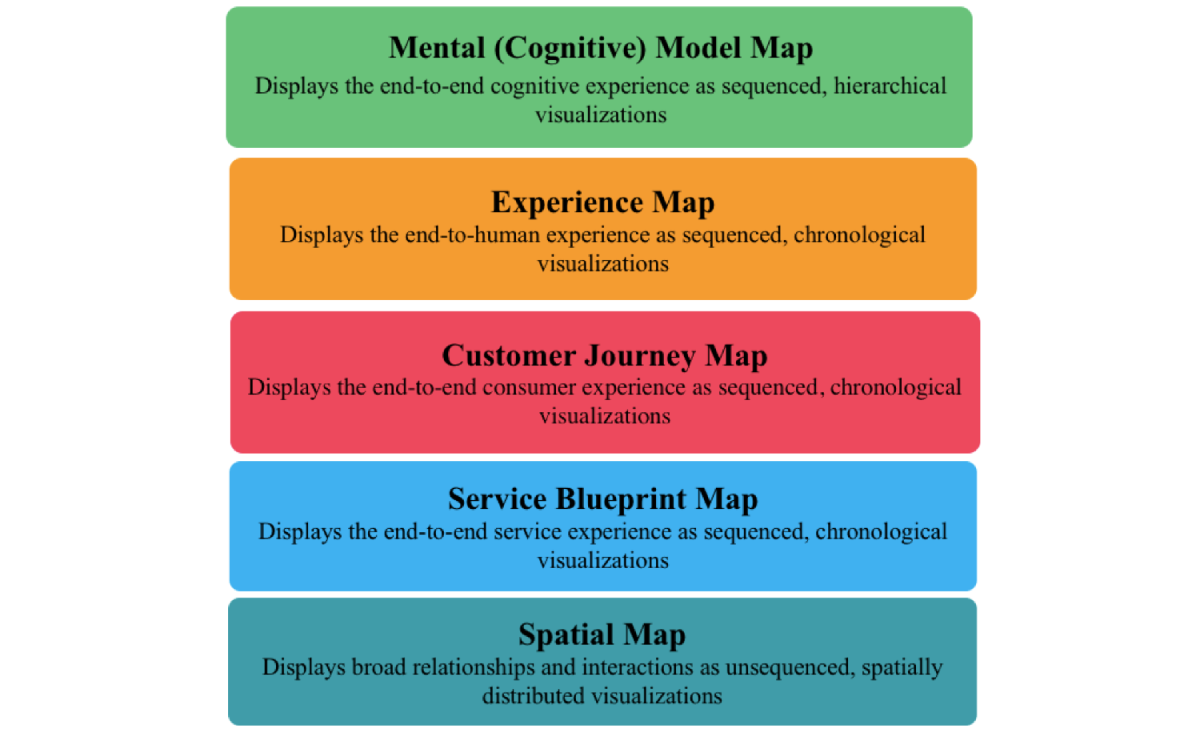
Five journey mapping techniques adapted from previous studies [1-3].

Journey maps are visualization techniques that can facilitate the diagrammatical representation of stakeholder groups by interest or function for comparative visual analysis [1,2]. Thus, in a health care context, journey maps can illustrate complex service delivery bottlenecks and describe the user experience across the continuum of care. There are 5 journey mapping techniques (Figure 1) that can each be used to illustrate a unique experience: (1) Mental (Cognitive) Model Map, (2) Experience Map, (3) Customer Journey Map, (4) Service Blueprint Map, and (5) Spatial Map [1-3]. Each mapping technique displays information distinctly and illustrates experiences in different contexts [1,2].

The benefit of these succinct visualizations lies in their ability to effectively illustrate intersections and relationships between organizations and consumers using products or services [4]. Therefore, journey maps can be used to help identify process pain points and highlight opportunities for improvement in various settings and contexts. Further, the visual findings of journey mapping activities can assist service providers and implementation scientists in effectively deploying resources to expand services or establish operational risks. As illustrated in Figure 1, the 5 journey mapping techniques have similarities and interrelationships yet provide distinct visual analyses [2]. Therefore, the sequence in which the mapping activities should be conducted depends on the intended outcome of the mapping exercise [2,5]. For example, the Mental (Cognitive) Model Map technique provides a visual analysis of the cognitive processes an individual may experience in their interactions with an activity, organization, or service [1-3]. The Experience Map technique displays the overall human experience of an individual’s activities not specific to an organization, product, or service [1-3]. Contrastedly, the Customer Journey Map technique illustrates a consumer’s interactions using a specific service, organization, or product [1-3]. Following this, the Service Blueprint Map technique illustrates experiences from a systems view [1-3] and relationships between organizational processes, individuals, and service delivery [1-3]. Lastly, the Spatial Map technique provides a broad view of relationships between processes, service delivery, and individuals [1-3].

### What is a Learning Health System?

A learning health system (LHS) is a concept that emerged from the Institute of Medicine’s Roundtable on Evidence-Based Medicine [6]. The vision of an LHS is to “generate and apply the best evidence for the collaborative health care choices of each patient and provider; to drive the process of discovery as a natural outgrowth of patient care; and to ensure innovation, quality, safety, and value in health care” [6]. Further, Rubin and Friedman describe the LHS “as the tapestry that emerges from weaving together efforts across: health information management, health IT, patient engagement, clinical care, research, and public health arenas aimed at utilizing data, information, and knowledge to improve health” [7]. Since its introduction in 2007, others have continued to adapt, redefine and expand on the concept and how it can be achieved. However, regardless of the varied definitions in the industry, the goals of an LHS are the same: “harness the power of data and analytics to learn from every patient, and feed the knowledge of ‘what works best’ back to clinicians, public health, and other stakeholders to create cycles of continuous improvement” [8].

### The Continuous Knowledge Translation Loop of an LHS

An LHS can be conceptualized as a continuous learning microcosm that uses various data streams in the health care sector to improve service delivery and the human experience. As the health sector is multifaceted, there is a tremendous opportunity to more effectively use the often-fragmented data (ie, data stored in siloed and disparate health information systems) globally. An important aspect of an LHS lies in its potential to facilitate a continuous cycle of learning using health care data [8]. The strategic use of such data could allow external evidence from studies, reviews, and trials to inform practice and enrich the evidence base and, ultimately, the health system [9]. Further, the data, serving as a continuous feedback loop, could foster a mechanism in which evidence-based practices could be effectively used across the care continuum to catalyze systemic industry change. Specifically, the data gleaned from continuous data feeds could be aggregated and leveraged to improve service delivery in clinical practice and improve patient outcomes.

### The Potential Value of Using Journey Map Data to Feed an LHS

As the health care sector operates on a 24/7 basis globally, an unquantifiable amount of data could be streamlined, examined, and used to improve efficiency in service delivery and holistically inform the health system. The fluid data cycle [8] outputs from each citizen (or patient), as they use various facets of the health system, could be captured and illustratively detailed via the 5 journey mapping techniques (Figure 1). Thus, the experiences of citizens and health care providers interfacing with the health system could be assessed and evaluated from multiple vantages and perspectives to inform the greater health ecosystem. Therefore, the data gleaned from the 5 journey mapping techniques [1-3] could provide a robust source and live data feed for a broader LHS and data repository. Additionally, integrating lived human experiences (ie, patient, physician, and caregiver journey mapping activities) into the design of health information systems (HIS) and health information technology (HIT) holds tremendous potential value for the creation of safer and more usable systems [10].

### Objective

This paper aims to conduct a scoping review assessing the current state of the literature to establish a relationship between journey mapping techniques and LHSs.

### Research Questions

- Is there a relationship between journey mapping techniques and an LHS in the literature?
- Is there a way to integrate the data from journey mapping activities into an LHS?
- How can the data gleaned from journey mapping activities be used to inform an LHS?

## Methods

**Figure 2:**
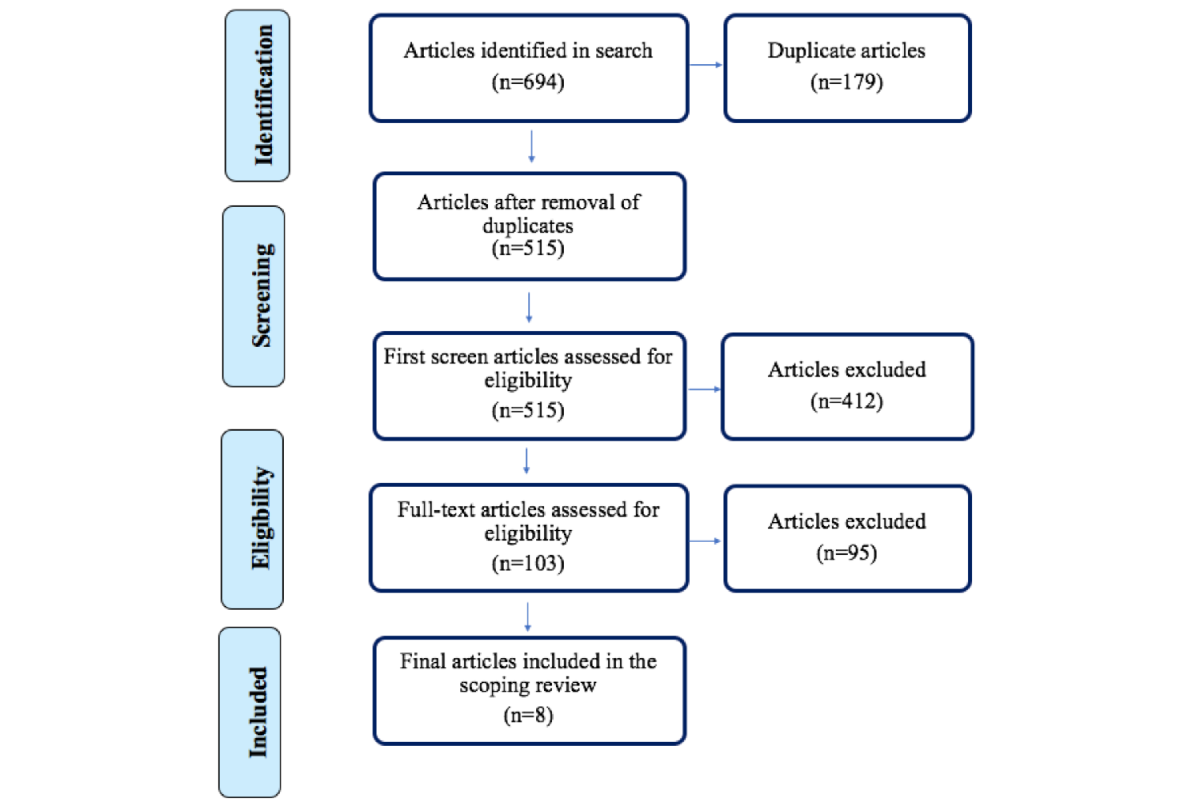
Adaptation of PRISMA (Preferred Reporting Items for Systematic Reviews and Meta-Analyses) data flow diagram detailing article selection during the screening process [12].

A scoping review, guided by the Arksey and O’Malley framework [11], was carried out by querying the following electronic databases: Cochrane Database of Systematic Reviews (Ovid), IEEE Xplore, PubMed, Web of Science, Academic Search Complete (EBSCOhost), APA PsycInfo (EBSCOhost), CINAHL (EBSCOhost), and MEDLINE (EBSCOhost).The key terms used were as follows: (Learning Health System) OR (Delivery of Healthcare), (Journey Mapping) OR (Patient OR Care) AND (Journey), and (Informatics) OR (Patient Health Information). The article evaluation began with a first screening in which 2 researchers independently assessed all articles by title and abstract using Covidence (Veritas Health Innovation), and articles were included (Figure 2) if they satisfied the following inclusion criteria:

- English articles with abstracts published between the years 2010 and 2022.
- Articles that referenced journey maps or mapping activities and an LHS.
- Articles that described user experiences in health care (eg, patients, caregivers, and physicians) and the LHS.

Subsequently, the 2 researchers independently screened and read the full-text articles to establish inclusion (Figure 2). Differences of opinion in article selection were resolved through discussion and team consensus. Lastly, the relevant data were extracted and tabulated for comparative analysis (Table 1), and the final selection of articles was assessed thematically to establish trends and themes in the literature.

**Table 1:** Data extraction table illustrating the themes represented by each paper in this scoping review.

Author	Study design	The need to evolve service delivery models in health care	The potential value of using patient journey data in an LHS^a^
Azar et al [13]	Descriptive	✓^b^	
Fung et al [14]	Pilot study	✓	
Gartner et al [15]	Concept analysis and systematic review	✓	
Sun et al [16]	Perspective	✓	
Yu [17]	Editorial	✓	
Joseph et al [1]	Scoping review		✓
Levine et al [18]	Pilot study		✓
Sharma et al [19]	Observational study		✓

^a^LHS: learning health system.

^b^✓: denotes the themes represented in each paper.

## Results

### Overview

The initial search yielded 694 studies. Of those, 179 duplicates were removed in Covidence. Following this, a first screening of the article sample was conducted, and 515 articles were assessed (Figure 2). During the first screening phase, 412 articles were excluded, as they did not meet the inclusion criteria. Next, a full-text review of all 103 remaining articles was done. Of those, 95 were excluded, resulting in only 8 relevant articles that satisfied the inclusion criteria.

### Thematic Analysis

After identifying relevant articles, each article was assessed thematically with data extracted and tabulated in Table 1. The findings from these articles can be subsumed into 2 overarching themes: (1) the need to evolve service delivery models in health care, which was expressed in 5 articles; and (2) the potential value of using patient journey data in an LHS, which was described in 3 articles. These 2 thematic categories will be examined in the subsequent sections.

#### Theme 1: The Need to Evolve Service Delivery Models in Health Care

With 5 articles stressing the urgency to evolve service delivery models in health care settings, it was the most prominent theme of the literature sample. In the article “The Indiana university center for healthcare innovation and implementation science: bridging healthcare research and delivery to build a learning healthcare system” [13], Azar et al detail that an “estimated 75,000 deaths every year could be prevented if high-quality care was more efficiently and effectively implemented” [13]. The authors quote the United States National Institute of Health, in that this considerable problem is not due to a paucity of knowledge, but rather poor incorporation of health care discoveries into daily practice [13]. Azar et al [13] clarify that over the past 3 decades, medical knowledge has increased, with 11 systematic reviews and 74 clinical trials being published every day, yet only 14% of these new findings are actually implemented in health care settings and translated into practice [13]. Therefore, to mitigate the risks to human health, the authors propose a paradigm shift in how health systems and service delivery should be conceptualized. They present 2 contrasting perspectives: (1) a traditional model of service delivery and (2) an innovative and adaptive model of health care service delivery. In the traditional model, organizations are viewed as machines that perform predictable, repeated tasks with replaceable parts that operate in stable and nondynamic settings [13]. In the adaptive model, health care systems are viewed as complex, dynamic, adaptive, and evolving systems comprised of a network of semiautonomous individuals (ie, health care professionals) who interact in nonlinear ways [13]. As health care needs and interactions are interdependent, interconnect, and changing over time [13], the authors insinuate that it is vital to design health care services to support the fluidity of systemic evolution. Thus, their article expressed the criticality of designing and developing an adaptable agile learning system that integrates hospital systems, population health, individual patients, and health care personnel [13].

Fung et al [14] present a systems approach to redesigning care in their article “Regional process redesign of lung cancer care: a learning health system pilot project.” Their novel approach enables timely access to cancer treatment for patients with lung cancer to a centralized specialty service that addresses clinical and operational challenges [14]. However, the authors caution that, despite its potential value, there is limited evidence of successful implementation of the LHS vision [14]. Thus, to streamline and operationalize the LHS concept, they developed the Ottawa Health Transformation Model as a regional approach to guide service delivery change and to integrate the nuances of the patient journey with best practices [14]. Further, the article laments that all facets of care need to be examined to address the complexity of health systems and to improve patient experiences holistically rather than just isolated parts [14]. The article concludes with the caveat that the value of the LHS approach in relation to service delivery is the creation of a system that can facilitate best practice adoption and fluid innovation [14]. Similarly, in their concept analysis and systematic review, Gartner et al [15] detail that a performant health care system is crucial for every country and that the current siloed health care business practices must be evaluated and challenged [15]. The authors suggest that fragmented health care services can compromise patient care, inhibit sustainable service delivery, and result in suboptimal use of financial and human resources [15]. Further, the authors state that repeated calls to improve the overall performance and quality of global health care delivery have occurred since 2001 [15]. The calls for transformational change in health care have been made by well-established national and international organizations such as the Institute of Medicine [15,20,21]; The National Academies of Sciences, Engineering, and Medicine [15,22]; and The World Health Organization [15,23,24]. Gartner et al [15] suggest that understanding the patient journey through an LHS view can facilitate the improvement of health care service delivery through a feedback loop in which data can be used to identify problem areas to support continuous improvement [15]. Lastly, in a similar yet contrasting view, Sun et al [16] express in their paper “Health management via telemedicine: learning from the COVID-19 experience” that telemedicine provides numerous opportunities to improve care efficiency, accessibility, and patient outcomes [16]. However, they state that many challenges exist, such as the digital divide, usability, and technology interoperability [16]. Further, the authors detail that the delivery of telemedicine services must evolve to support continuity of care throughout the patient journey [16]; specifically, by including the seamless integration of data from the clinical workflow of multidisciplinary care teams to support the LHS [16]. Nonetheless, they clarified that the implementation of a telemedicine business model must be supported by rigorous evidence-based practices, including clinical trials [16]. They warned that such precautionary measures are necessary to facilitate the seamless integration of telemedicine into routine care, ensuring the quality and safety of virtual care delivery [16]. Lastly, Yu et al [17] recount that data are only important and useful when they can be transformed into knowledge. In a health care context, the importance of data is realized when data sets of individual patients can be aggregated with similar patient data to inform patient populations [17]. Further, the value of clinical data lies in its interpretation in a clinical context among continuing care providers and when it is shared with the patient or their caregivers [17]. Additionally, the data set of a citizen (ie, patient) becomes of greater importance when it is combined with that of other citizens and when it can be aggregated for comparative statistical analysis to inform the health system on the health status of a population or subset [17].

#### Theme 2: The Potential Value of Using Patient Journey Data in an LHS

The potential value of using patient journey data in an LHS was expressed in 3 articles. In the article “Patient journey mapping: current practices, challenges and future opportunities in healthcare,” Joseph et al [1] describe how the data gleaned from patient journey maps could improve the health system by identifying varying patient experiences. Additionally, Joseph et al [1] detail that journey mapping approaches hold a significant value in improving complex health care processes for patients and providers alike. Further, the authors express that closely integrating patient journey mapping techniques into the health care system could create an LHS [1]. In their study “Learning health system for breast cancer: pilot project experience,” Levine et al [18] report that clinicians need accurate and timely information on patient outcomes associated with various treatment modalities. Moreover, the authors describe that electronic health records are perceived to be helpful technologies, but access to patient data is often difficult [18]. However, despite the data accessibility challenges expressed in their study, the researchers were able to combine, read, and extract electronic health records data to view the patient journey [18]. Specifically, Levine et al [18] developed a prototype leveraging IBM Watson technology, with capabilities to validate artificial intelligence using natural language processing and to denote the clinical course of patients (ie, patient journey) in support of an LHS platform [18]. Their study findings illustrated a means by which the vision of an LHS could potentially be achieved by using artificial intelligence [18]. Despite the preliminary nature of their study, the authors were able to demonstrate that the hospital had the necessary data to formulate a view of the patient journey, which could be extracted and used in ways to support clinical decision-making [18]. Lastly, in their observational study, Sharma et al [19] used an incremental and iterative approach, engaging administrative and clinical domain experts to demonstrate that human actors, rather than IT, are the central focus of data movement [19]. The authors evaluated a kidney transplant referral pathway and established the relationship between human actors, organizations, the complexity of data administration, and data flow bottlenecks [19]. Their study illustrated the manual and often cumbersome tasks that clinical staff must perform to access and visualize health data from fragmented IT systems [19]. The authors express broadly that IT systems that are not interoperable can lead to data access challenges and complicate the clinical workflow and health care providers’ ability to effectively and efficiently perform their job functions [19]. They further reveal that in a kidney transplant referral context the lack of centralized and timely access to patient data can delay patients’ registration on the transplant list, as the time and effort to complete referral forms are greatly increased [19]. Sharma et al [19] propose that an LHS with linked patient data can improve population health outcomes and inform interventions by providing timely and intuitive access to health information.

### Summary of Findings

Despite the comprehensive search, the research questions were only partly satisfied. The first research question, “Is there a relationship between journey mapping techniques and an LHS in the literature?” was demonstrated in both thematic categories. There is a relationship and a need for an innovative approach to health care design and service delivery. As shown in Table 1, five articles exemplify the need to evolve service delivery models in various scenarios in health care. Three articles provide insight into the potential value of using patient journey data to inform an LHS. The second research question, “Is there a way to integrate the data from journey mapping activities into an LHS?” was not comprehensively addressed, and an actionable, scalable plan was not provided in the literature. The third question, “How can the data gleaned from journey mapping activities be used to inform an LHS?” was satisfied by the scoping review findings (Table 1). Many articles provided examples of operational gaps and scenarios in which patient care could be compromised due to a lack of timely, interoperable, and accessible data.

## Discussion

### Principal Findings

This study has presented a scoping review using articles from the following electronic databases: Cochrane Database of Systematic Reviews (Ovid), IEEE Xplore, PubMed, Web of Science, Academic Search Complete (EBSCOhost), APA PsycInfo (EBSCOhost), CINAHL (EBSCOhost), and MEDLINE (EBSCOhost). As evidenced by the PRISMA (Preferred Reporting Items for Systematic Reviews and Meta-Analyses) diagram (Figure 2), of 694 initially screened articles, only 8 satisfied the inclusion criteria. Within the articles that met the inclusion criteria, we identified 2 important themes: (1) five articles stressed the need to evolve service delivery models in health care, and (2) three articles described the potential value of using patient journey data in an LHS. Despite the robust search strategy and databases used, there was a dearth of literature discussing a relationship between journey mapping and LHSs. Therefore, the first research question, “Is there a relationship between the journey mapping techniques and an LHS in the literature?” was only partially satisfied. Despite the included articles [1,13-19] providing various scenarios and applications of the relationship potential and how journey mapping could support value-based and patient-centric care strategies for LHSs [25], more research is required in this arena. Further, given the current state of the literature, we could not address the second research question, “Is there a way to integrate the data from journey mapping activities into an LHS?” Although the urgency of timely access to centralized, high-quality, interoperable data was prominent in the literature, a comprehensive road map or framework was not provided to integrate the data specifically from journey mapping activities into an LHS. Lastly, the third question, “How can the data gleaned from journey map activities be used to inform an LHS?” was satisfied by the scoping review findings (Table 1). Many articles provided examples of operational gaps and scenarios in which patient care could be negatively impacted by workflow bottlenecks or disruptive technologies. However, specifically how patient journey map data could be used to inform the continuous learning feedback loop of an LHS, which could inform evidence-based practices, was not provided. Further, the article sample did not provide detail on how the 5 journey mapping techniques (Figure 1) could independently or collectively provide rich and diverse continuous data supply (ie, a continuous knowledge translation loop) for an LHS.

To address the shortcomings in the literature, the authors will continue this line of investigation to establish a relationship between the concept of an LHS and the 5 journey mapping techniques: (1) Mental (Cognitive) Model Map, (2) Experience Map, (3) Customer Journey Map, (4) Service Blueprint Map, and (5) Spatial Map [1-3]. Specifically, this scoping review will be phase 1 of an investigative series. Phase 2 will entail the creation of a holistic framework to guide and streamline data integration from journey mapping activities outputs into an LHS. Lastly, phase 3 will provide a proof of concept to demonstrate how journey mapping activities could be integrated into an LHS.

### Limitations

As this is a preliminary study, the scoping review was limited to only digital articles in English; therefore, other relevant articles could have been omitted based on the study design. Future studies could include paper-based searches and searches in other languages. Moreover, journey maps are not widely or consistently used in the health care sector [1,2], which may have contributed to the study’s small sample of only 8 relevant articles. Similarly, although poised to improve health care sustainably through smart and efficient data use, LHS is a relatively novel and emerging concept in the health care landscape [6].

### Conclusions

This paper expressed the criticality and urgent need of global health care transformation to support the sustainable delivery of health care services. Additionally, it was revealed that current health systems are not adequately using the health data in which they aggregate institutionally. Consequently, fragmented and siloed data are stored in disparate HIS and HITs on a global scale. Thus, there is a dire need to design and develop an agile and interoperable LHS that can integrate global data from health care organizations, populations (ie, citizens, patients, caregivers, physicians, and health care stakeholders), HIS, and HIT. Journey mapping activities provide an opportunity and a conduit to streamline data into uniform and usable formats. Thus, the knowledge gap related to integrating the data from journey mapping activities into an LHS highlighted the importance of using the data from patient experiences to enrich an LHS and provide holistic care. Moreover, the journey mapping visualizations of the 5 mapping techniques (Figure 1) could identify operational issues such as staffing shortages, clinical workflow bottlenecks, and other factors that could negatively impact patient care [1,2]. The visualizations could also illustrate scenarios where health care service design and delivery could be stifled or affected from a clinical lens by physician burnout and cognitive impairment from alert fatigue [26]. Integrating the data from the 5 journey map techniques [1-3] into an LHS promises to improve health care service delivery and patient outcomes by providing a continuous supply of data to support patient-centric health care solutions that meet the goals of patients and providers.

## Abbreviations

HIS: health information systems
HIT: health information technology
LHS: learning health system
PRISMA: Preferred Reporting Items for Systematic Reviews and Meta-Analyses
